# Distinct Regulatory Mechanisms Act to Establish and Maintain Pax3 Expression in the Developing Neural Tube

**DOI:** 10.1371/journal.pgen.1003811

**Published:** 2013-10-03

**Authors:** Steven Moore, Vanessa Ribes, Javier Terriente, David Wilkinson, Frédéric Relaix, James Briscoe

**Affiliations:** 1 MRC-National Institute for Medical Research, Medical Research Council, London, United Kingdom; 2 Myology Group, UMR-S787 INSERM, Université Pierre et Marie Curie Paris VI, Pitié-Salpétrière, Paris, Paris, France; New York University, United States of America

## Abstract

Pattern formation in developing tissues is driven by the interaction of extrinsic signals with intrinsic transcriptional networks that together establish spatially and temporally restricted profiles of gene expression. How this process is orchestrated at the molecular level by genomic cis-regulatory modules is one of the central questions in developmental biology. Here we have addressed this by analysing the regulation of Pax3 expression in the context of the developing spinal cord. Pax3 is induced early during neural development in progenitors of the dorsal spinal cord and is maintained as pattern is subsequently elaborated, resulting in the segregation of the tissue into dorsal and ventral subdivisions. We used a combination of comparative genomics and transgenic assays to define and dissect several functional cis-regulatory modules associated with the Pax3 locus. We provide evidence that the coordinated activity of two modules establishes and refines Pax3 expression during neural tube development. Mutational analyses of the initiating element revealed that in addition to Wnt signaling, Nkx family homeodomain repressors restrict Pax3 transcription to the presumptive dorsal neural tube. Subsequently, a second module mediates direct positive autoregulation and feedback to maintain Pax3 expression. Together, these data indicate a mechanism by which transient external signals are converted into a sustained expression domain by the activities of distinct regulatory elements. This transcriptional logic differs from the cross-repression that is responsible for the spatiotemporal patterns of gene expression in the ventral neural tube, suggesting that a variety of circuits are deployed within the neural tube regulatory network to establish and elaborate pattern formation.

## Author Summary

The complex organization of tissues is established precisely and reproducibly during development. In the vertebrate neural tube, as in many other tissues, the interplay between extrinsic morphogens and intrinsic transcription factors produces spatial patterns of gene expression that delineate precursors for specific cell types. One such transcription factor, Pax3, defines the precursors of all sensory neuron subtypes and distinguishes them from precursors fated to give rise to the motor circuits. To gain insight into the molecular mechanisms by which the spinal cord is segregated into these two functional domains, we analysed the genomic regulatory sequences responsible for controlling Pax3 activity. We identified two regions of the genome, the coordinated activity of which establishes and refines Pax3 activity. We showed that the combination of activating signals from secreted Wnt factors together with Nkx family homeodomain repressors restrict Pax3 activity to the presumptive sensory region of the neural tissue. Subsequently, Pax3 acts to directly potentiate its own transcription and this autoregulation sustains Pax3 expression at later developmental stages. Together, our study reveals the way in which intrinsic and extrinsic signals are integrated by cells and converted into a sustained pattern of gene activity in the developing nervous system.

## Introduction

Embryonic development relies on the coordinated and dynamic control of gene expression. This is achieved, in the main, by interactions between transcription factors (TFs) and the genomic cis-regulatory modules (CRMs) associated with regulated genes [1], [2]. The aggregate of these interactions produces a gene regulatory network (GRN) that is responsible for imparting distinct molecular identities and consequently the pattern of cell fate in a tissue. Within these large GRNs, sub-circuits can be discerned that confer specific behaviors and responses [1], [3]. Thus, elucidating functional interactions between TFs and CRMs provides insight into the mechanism and regulatory logic of the transcriptional networks responsible for tissue patterning.

The specification of progenitor identity in the vertebrate neural tube is a well studied example of developmental patterning [4]. Motor neurons and several classes of associated interneurons are generated in ventral regions of the neural tube in response to the morphogen Sonic hedgehog (Shh). Secretion of Shh from the notochord and floor plate establishes a gradient of intracellular signaling activity that regulates the expression of TFs specifying the ventral progenitor domains [5]–[8]. Key phylogenetically conserved CRMs associated with many of these TFs have been identified and shown to integrate the activity of Shh signaling with general neural TFs and Shh regulated TFs [9], [10]. Within this network selective cross-repressive interactions between TFs operating downstream of Shh signaling appear critical, both to establish and maintain the distinct spatial domains of progenitors [5], [6], [11]–[14].

By contrast, less is known regarding the specification of sensory interneurons within the dorsal spinal cord [15]. One key TF involved in this process is the paired homeodomain protein Pax3, which is amongst the first to delineate the dorsal neural tube and then later, together with its paralog Pax7, identifies the 6 progenitor domains that comprise dorsal progenitors [16], [17]. Both bone morphogenetic protein and Wnt signaling have been implicated in the induction of Pax3 transcription and the establishment of dorsal progenitors [18], [19]. Conversely, Shh mediated repression of Pax3 has been suggested to eliminate expression in the ventral neural tube 20,21.

Several studies indicate that a genomic interval immediately upstream of the mouse Pax3 promoter is sufficient to direct expression to the neural tube, however this region is not required for Pax3 expression [22]–[25]. A further two CRMs have been identified within the 4th intron that recapitulate elements of Pax3 expression in the central nervous system (CNS) [22], [26]. The activity of one region, termed ECR2 [22] or IR1 [26], is dependent on Tcf/Lef binding sites, consistent with a role for Wnt signaling in the initiation of Pax3 transcription. Nevertheless, how the spatial domain of Pax3 expression is determined and maintained during the elaboration of neural tube patterning has not been explained.

Here we take advantage of lineage tracing analyses in mice and transgenic assays in chick and zebrafish embryos to dissect the molecular mechanism and regulatory logic of Pax3 expression in dorsal neural progenitors. We show that Pax3 expression is refined during neural tube patterning by the temporal activity of distinct regulatory elements. We provide evidence that in addition to Wnt signaling, Nkx family homeodomain (HD) containing repressors are critical for establishing the restricted expression of Pax3. Moreover, we demonstrate that autoregulation and positive feedback is required to maintain Pax3 expression in the neural tube.

## Results

### The dynamics of Pax3 expression in the neural tube is recapitulated by 2 CRMs

**Figure 1:**
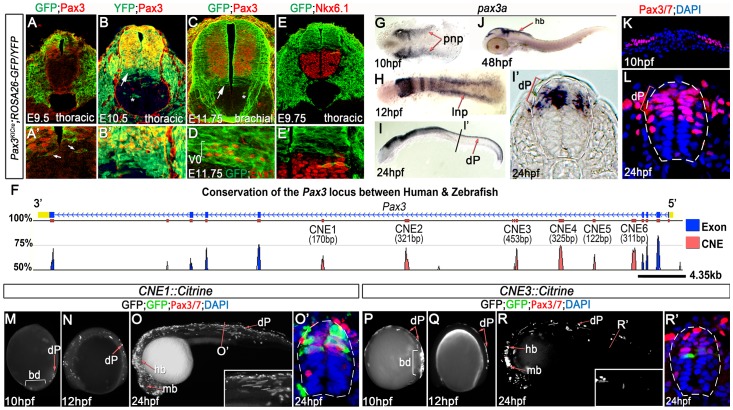
Pax3 expression is recapitulated by the activity of 2 CRMs. (A, A′) At E9.5, Pax3 expression correlates with cells labeled by YFP in *Pax3^Cre/+^;Rosa26-YFP* mouse embryos (n = 2). However between E10.5 (B) (n = 2) and E11.75 (C) (n = 2), cells derived from the Pax3 lineage are found in the both the dorsal and ventral neural tube of *Pax3^Cre/+^;Rosa26-GFP/YFP* mouse embryos. In the ventral neural tube, clusters of isolated cells (asterisk in B and C) and a domain spanning 3–4 cells adjacent to the Pax3 ventral boundary (B′ and arrows in B and C) is observed. (D) This domain of transgene expression encompasses, but is not limited to, Evx1 expressing ventral interneurons at E11.75 (n = 2). (E, E′). Consistent with the labeling of interneurons at E11.75, embryos assessed at E9.75 exhibit a boundary of Pax3 lineage opposed to the dorsal limit of the Nkx6.1 domain (n = 3). (F) Comparative genomic analysis of the Pax3 locus reveals 6 CNEs within the 4^th^ intron of the gene. (G) *Pax3a* mRNA is first detected in the developing midbrain, hindbrain and intermediate regions of the posterior neural plate (pnp) of zebrafish embryos at 10 hpf. (H) By 12 hpf, transcription is limited to the lateral neural plate (lnp) posteriorly. (I) At 24 hpf, *pax3a* is highly expressed in the midbrain, hindbrain and progenitors within the dorsal spinal cord (dP), as shown in (I′). (J) Transcription is rapidly downregulated in the spinal cord after 24 hpf and is not detectable at 48 hpf. (K–L) Pax3/7 protein, visualised with the DP312 antibody, is also restricted to the intermediate and lateral regions of the neural plate at 10 hpf and the dorsal half of the spinal cord at 24 hpf. (M–N) Profile views of *CNE1::Citrine* transient transgenic embryos at 10 and 12 hpf showing enhancer activity in the tail bud (bd) and lateral neural plate (M) (n = 25/27), then within dorsal neural progenitors (N) (n = 19/22). (O, O′) At 24 hpf, *CNE1::Citrine* transient transgenics recapitulate *pax3a* expression across the AP axis of the CNS and the DV axis of the spinal cord. (P–R′) CNE3 is active in the neural plate and tail bud at 10 and 12 hpf (n = 10/10, n = 7/7), however embryos rarely exhibit labeling of Pax3/7 expressing progenitors within the dorsal spinal cord at 24 hpf (n = 5/48).

The establishment of the Pax3 expression domain in the neural tube distinguishes the progenitors of sensory neurons from those fated to give rise to motor neurons and associated ventral interneurons, however the cellular and molecular mechanisms that regulate this key patterning event remain poorly understood. In order to gain insight into this process, we employed a lineage tracing approach to assay the spatiotemporal dynamics of Pax3 expression in the neural tube. Transgenic mice in which Cre recombinase was targeted to the first exon of the Pax3 locus [27] were crossed with either *Rosa26-YFP* or *Rosa26-Tomato/GFP* reporter strains (Figure 1A–E′ and data not shown). The resulting embryos were analysed between embryonic days (E) 8.5 and E11.75 in transverse sections. From E8.5 to E9.5, all cells marked by transgene expression also express Pax3, demonstrating that this transgenic line accurately reports the Pax3 lineage (Figure 1A, A′ and data not shown). At these early stages the Pax3 expression domain is not well defined and isolated cells expressing both GFP and Pax3 can be detected within the intermediate region of neural tube (arrows in Figure 1A′). From E9.5 onwards transgene labelled cells were observed beyond the ventral boundary of Pax3, indicating that the position of the Pax3 domain was refined during early stages of neural tube patterning (Figure 1B, B′ and C).

Two distinct populations of transgene labelled progenitors that no longer express Pax3 protein were present within the ventral neural tube of each embryo. The first comprised isolated clusters of cells (asterisks in Figure 1B). The dispersal of these cells within the ventral neural tube was highly variable, both between stage-matched siblings and along the anterior-posterior (AP) axis of individual embryos. We attributed this to the induction of Pax3 transcription within the neural plate and cell mixing within the neuroepithelium at early stages of development [28]–[30]. By contrast, the second population of transgene expressing cells was a continuous domain that spanned 3–4 cell diameters adjacent to the ventral boundary of Pax3 (Figure 1B′ and arrows in B and C). The extent of transgene expression encompassed, but was not limited to, the Evx1 expression domain across the AP axis of embryos assessed at E11.75 (Figure 1D). These data indicated that cells fated to become ventral interneurons extinguish Pax3 expression during early CNS patterning [31]. In agreement with this observation, the Pax3 lineage apposed Nkx6.1 expression at E9.75, which labels the 3 most ventral progenitor domains of the neural tube (Figure 1E and E′). Together, these data demonstrated that the initial domain of Pax3 expression was refined by a switch in progenitor identity and subsequently maintained at this DV position.

We next sought to investigate the molecular basis of Pax3 expression during CNS development by employing comparative genomics to identify functional CRMs associated with the gene. Conservation of the Pax3 locus and the surrounding intergenic regions across the human, mouse, zebrafish and fugu genomes revealed 6 conserved non-coding elements (CNEs), all of which were located within the 4^th^ intron of the gene (Figure 1F and Figure S1). Candidate CRMs were assayed in zebrafish embryos, which have been previously shown to exhibit similar spatio-temporal profiles of *pax3a* mRNA expression to that observed in chick and mouse [16], [20], [32]. Accordingly, *pax3a* expression was first observed in the posterior neural plate at 10 hours post fertilisation (hpf) (Figure 1G) before becoming restricted to the lateral limit of the tissue at 12 hpf (Figure 1H). Maximum expression within the dorsal neural tube was observed at 24 hpf (Figure 1I and I′), after which transcription rapidly decreased to undetectable levels in the spinal cord by 48 hpf (Figure 1J and data not shown). Pax3 and Pax7 (Pax3/7) proteins were expressed in the lateral regions of the posterior neural plate at 10 hpf (Figure 1K) and progenitors within the dorsal spinal cord at 24 hpf (Figure 1L), in agreement with *pax3a* transcription. Strikingly, each CNE assayed in transient transgenic zebrafish exhibited a tissue specific enhancer activity at 24 hpf, the majority of which recapitulated elements of *pax3a* expression (Figure S1). However, only CNE1 and CNE3 reproducibly labelled the developing CNS (Figure 1M–R′).

**Figure 2:**
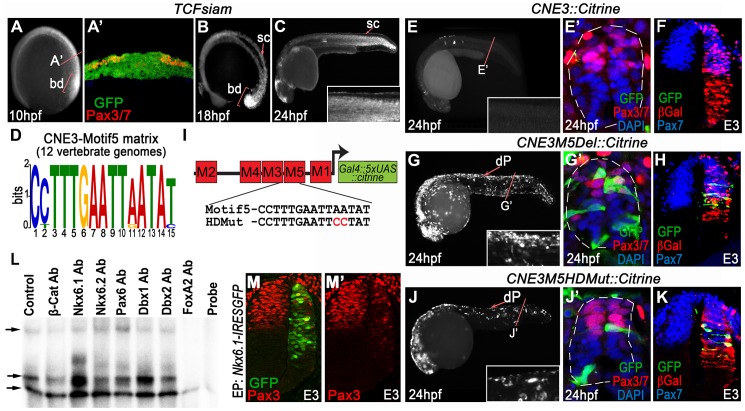
CNE3 balances transcriptional activation and repression to establish the Pax3 expression domain. (A) *TCFsiam* transgenic zebrafish assessed at 10 hpf reveal activated Wnt signaling in the posterior neural plate and tailbud (bd). (A′) Transverse sections of the posterior neural plate demonstrate that Wnt pathway activity is not restricted in the medio-lateral axis of the tissue, whereas Pax3/7 is expressed laterally. (B–C) Wnt signaling is maintained within the neural tube up to 18 hpf, following which it rapidly declines and is found only in the dorsal most row of cells within the neural tube at 24 hpf. (D) Matrix indicating the conservation of CNE3-Motif5 across 12 vertebrate genomes. (E, E′) Zebrafish injected with a CNE3 reporter rarely contain labeled cells within the dorsal spinal cord of transient transgenic embryos at 24 hpf (n = 4/33) and the reporter is not active in the chick neural tube at E3 (F) (n = 0/7). (G, G′) *CNE3Motif5Del::Citrine* transgenics exhibit an increase of enhancer activity across the DV axis of the spinal cord, compared to controls (n = 28/32, p<0.0001). (H) Furthermore, deletion of Motif5 results in the ectopic induction of CNE3 activity in chick embryos at E3 (n = 4/4, p = 0.003). (I) Schematic depicting the organisation of motifs within CNE3 and the mutation induced within the conserved HD binding site in Motif5. (J, J′) Mutation of the HD binding site in Motif5 increases CNE3 activity in progenitors within the zebrafish spinal cord, compared to controls (n = 21/21, p<0.001). (K) Chick embryos electroporated with *CNE3M5HDMut* DNA exhibit ectopic enhancer activity in the developing neural tube (n = 3/4, p = 0.0242). (L) EMSAs performed using a DNA probe spanning Motifs 3–5 of CNE3 and chick spinal cord nuclear extract. Complexes formed with nuclear extract are indicated (arrows, compare Control to Probe lanes). Addition of an Nkx6.1 antibody creates a slower migrating DNA/protein complex than controls, whereas addition of FoxA2 antibody abrogates complex formation. Both results indicate that these proteins are able to bind CNE3. Addition of Dbx1, Dbx2, Pax6 and Nkx6.2 antibodies have a minor effect on the motility of EMSA complexes. (M, M′) Consistent with binding and mutagenesis studies, the electroporation of Nkx6.1 in the chick neural tube is sufficient to repress endogenous Pax3 protein expression in chick embryos (n = 11).

The genomic interval corresponding to CNE3 is a highly conserved region of a previously defined CNS specific Pax3 CRM, termed both ECR2 [22] and IR1 [26]. CNE3 transgenic embryos assessed between 10 and 24 hpf exhibited reporter expression in the posterior neural plate and neural rod between 10 and 18 hpf (Figure 1P, Q and data not shown). The developing midbrain and hindbrain were labeled at 24 hpf, however transgene expression within dorsal spinal cord progenitors was markedly reduced by this stage (Figure 1R, R′, 2E, E′ and Figure S1). Together, these data suggested that the profile of CNE3 activity correlated with the induction of Pax3 transcription and the establishment of this expression domain. However, the down regulation of CNE3 activity in spinal cord progenitors indicated that a separate CRM was required to maintain high levels of Pax3 at later developmental stages.

In addition to CNE3, our functional assays identified CNE1 as a CNS specific Pax3 enhancer. CNE1 transient transgenic zebrafish exhibited Citrine expression in presumptive dorsal progenitors at 10 hpf (Figure 1M) and 12 hpf (Figure 1N). Robust labeling of the midbrain, hindbrain and dorsal spinal cord progenitors was observed at 24 hpf (Figure 1O, O′ and Figure S1). These data were supported by the creation of 3 independent CNE1 stable lines, which revealed that the activity of this CRM recapitulated Pax3 expression during the first 24 hours of CNS development (Figure S2A–B′). Moreover, CNE1 activity was restricted to the Pax3/7 domain of the zebrafish neural tube at 24 hpf (Figure 1O′ and Figure S2B′). These findings were consistent with reports documenting the activity of an approximately 2.5 kilobase genomic interval containing this enhancer, named IR2, in zebrafish embryos [26]. These data suggested that CNE1 might act in concert with CNE3, initially to define the Pax3 domain in the neural plate and later function independently to maintain expression in the neural tube. This hypothesis was supported by examining the binding profile of the transcriptional coactivator p300 in E11.5 mouse tissue, which suggested that CNE1 was the only active enhancer within the Pax3 locus at this comparatively late stage of CNS patterning (Figure S3) [33].

### CNE3 mediates the induction of Pax3 transcription

We sought to investigate the molecular basis of CNE3 activity in order to gain insight into the regulation of Pax3 expression in the CNS. The conservation of sequence across 12 vertebrate genomes was used to define 5 statistically enriched 15 bp motifs, predicted to contain the transcription factor binding sites (TFBS) that mediate enhancer activity (Table S1 and Figure S4). Annotation of TFBS within Motif4 and Motif5 of CNE3 suggested that these regions contained highly conserved Tcf/Lef binding sites (Table S1), consistent with reports demonstrating the requirement of Tcf/Lef sites within this CRM [22], [26]. Furthermore, the Wnt pathway effector Tcf3 has been shown to bind this enhancer in ChIP-Seq experiments performed in mouse embryonic stem cells [34] (Figure S5). These data suggested that CNE3 received positive transcriptional input from the Wnt pathway, which has been shown to be both necessary and sufficient for the induction of Pax3 transcription [18].

We used *TCFSiam* transgenic zebrafish [35] to assay the transcriptional activity of the Wnt pathway at 10 hpf, the stage at which CNE3 activity and Pax3 expression was first detected (Figure 2A, A′). This revealed activated Wnt signaling throughout the medio-lateral axis of the posterior neural plate (Figure 2A and A′), whilst Pax3 expression was restricted laterally (Figure 2A′). This profile of activated Wnt signaling was consistent with that described in the neural plate of independent zebrafish [36] and mouse transgenic reporters [37]–[39]. These data supported the described role of the Wnt pathway in the initiation of Pax3 transcription, but also suggested that it could not provide sufficient positional information to establish the domain of Pax3 expression in the neural plate. *TCFSiam* transgenic embryos assessed between 12 hpf and 18 hpf exhibited activated Wnt signaling within the tailbud and neural tube, however reporter expression was markedly decreased in the spinal cord of 24 hpf embryos (Figure 2B, C and data not shown). This temporal profile of activated Wnt signaling correlated with the activity of CNE3 during embryogenesis (Figure 1P–R′ and data not shown), supporting a positive transcriptional role for Wnt pathway effectors upon CNE3.

We next searched for conserved TFBS that could facilitate the binding of putative repressors to CNE3, which might act to prohibit Pax3 transcription in the presumptive ventral neural tube. We focused upon Motif3 and Motif5, as these matrices exhibited homology to sites bound by HD and Fox family transcription factors (Figure 2D and Table S1). We were particularly intrigued by the identification of highly conserved Nkx binding sites within CNE3 as members of this gene family are key fate determinants within the ventral neural tube, functioning as repressors via recruitment of Groucho/Tle proteins [40].

We deleted either Motif3 or Motif5 from CNE3 and assayed enhancer activity in chick and zebrafish. Deletion of Motif3 resulted in an increase in the number of progenitors with reporter activity within the zebrafish spinal cord, however this effect was not observed in chick embryos (data not shown). By contrast, deletion of Motif5 resulted in a significant increase in CNE3 activity along the entire D–V axis of the zebrafish spinal cord (compare Figure 2G, G′ to Figure 2E, E′). Ectopic activation of the zebrafish enhancer sequence was also seen within the intermediate neural tube of chick embryos, in a domain adjacent to the Pax3 ventral boundary (Figure 2H), compared to the wildtype sequence (Figure 2F). Targeted substitutions were then engineered into CNE3 that mutated the HD binding site within Motif5 (Figure 2I). This mutation resulted in an increase of CNE3 activity in neural progenitors within the zebrafish spinal cord at 24 hpf (Figure 2J, J′) and the ectopic activation of CNE3 in the chick neural tube at E3 (Figure 2K), recapitulating the effect of Motif5 deletion.

To investigate the binding of putative repressors to CNE3, we performed electrophoretic mobility shift assays (EMSAs) using nuclear extracts from dissected E3 chick spinal cords and a 48 bp DNA probe which spanned Motif3, 4 and 5. Supershift reactions were performed by the addition of antibodies raised against β-catenin, several candidate HD containing ventral fate determinants and FoxA2 (Figure 2L and data not shown). Assays containing labeled probe and nuclear extracts led to the formation of 3 distinct complexes, compared to reactions in which the nuclear extract was omitted (compare the Control and Probe lanes in Figure 2L). Addition of Nkx6.2, Dbx1, Dbx2 and Pax6 antibodies resulted in some reduction in the motility of the second complex (Figure 2L and data not shown), however this effect was substantially more pronounced in reactions containing Nkx6.1 antibody. Addition of β-catenin antibody did not affect the motility of complexes. Furthermore, the addition of an antibody raised against FoxA2 completely blocked the formation of the 3 DNA/protein complexes (Figure 2L). These assays indicated that both Nkx6.1 and FoxA2 were present within DNA bound complexes, consistent with the annotation of conserved binding sites within the motifs that comprise this region (Figure 2D and Table S1).

Together, these data suggested that Nkx6.1, or a protein with a similar binding specificity, interacted with Motif5 of CNE3 to mediate transcriptional repression of Pax3 within the developing spinal cord. This is consistent with the observation that the ventral domain of the Pax3 lineage was mutually exclusive with Nkx6.1 expression in transgenic mice assessed at E9.75 (Figure 1E and E′). Furthermore, overexpression of Nkx6.1 in chick embryos repressed endogenous Pax3 protein expression in the dorsal neural tube at E3 (Figure 2M and M′). Thus, these data support a model in which CNE3 functions to establish the Pax3 domain in the posterior neural plate by integrating a positive input from the Wnt pathway and Nkx family transcriptional repressors.

### CNE1 maintains Pax3 expression by autoregulation and positive feedback

**Figure 3:**
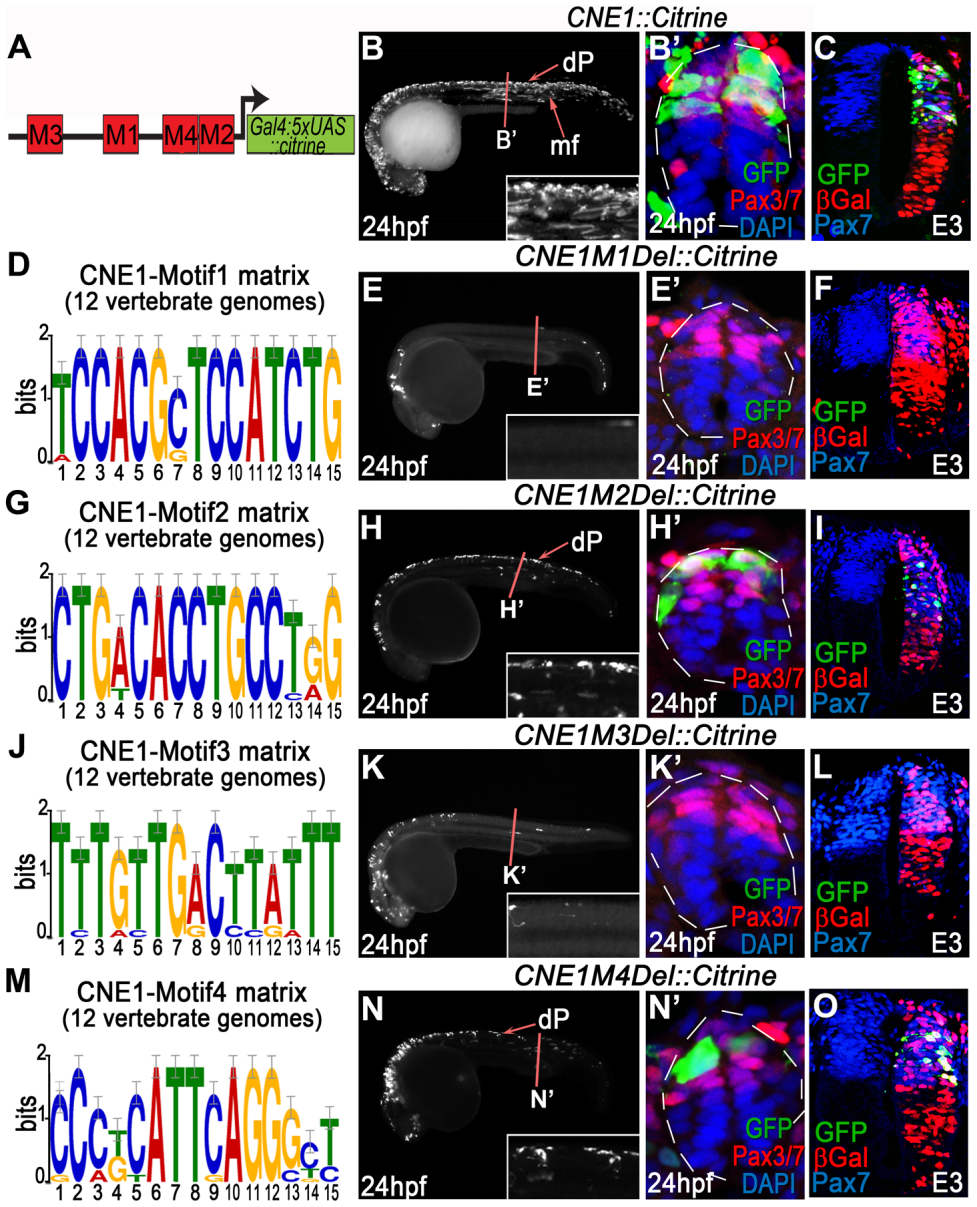
Functional dissection of CNE1 enhancer activity. (A) Schematic outlining the organisation of motifs within CNE1. (B, B′) CNE1 transient transgenic zebrafish embryos recapitulate *pax3a* expression across the AP axis of the CNS, including the spinal cord (n = 66/67). (C) Zebrafish CNE1 sequence specifically drives transgene expression in the Pax3/7 domain of the chick spinal cord, despite widespread transfection of LacZ across the DV axis of the tissue (n = 10/12). (D) Matrix representing the degree of Motif1 conservation across 12 vertebrate genomes. (E, E′) Deletion of Motif1 results in a complete loss of CNE1 activity in zebrafish spinal cord at trunk level (n = 0/22, p<0.0001), however the enhancer remains active in the anterior CNS (n = 22/22) and the most posterior region of the spinal cord (n = 8/22). (F) Loss of Motif1 greatly reduces CNE1 activity in the chick neural tube (n = 1/8, p = 0.0019). Motif2 (G), is not required for CNE1 activity in the zebrafish (H, H′) (n = 37/38) or chick (I) (n = 4/6) spinal cord. Loss of Motif 3 (J) reduces CNE1 mediated transcription across the AP axis of the zebrafish CNS (K, K′) (n = 0/26, p<0.0001) and precludes activity in the chick spinal cord (L) (n = 0/5, p = 0.003). Deletion of Motif4 (M) does not significantly alter the activity of CNE1 in zebrafish (N, N′) (n = 20) or chick (O) (n = 4/5).

We next sought to investigate the molecular basis of CNE1 activity by identifying statistically over represented conserved 15 bp motifs within this ∼170 bp enhancer (Figure 3A, Table S2 and Figure S6). Control experiments demonstrated that CNE1 activity was restricted to the Pax3/7 domain of the dorsal spinal cord in both zebrafish embryos at 24 hpf (Figure 3B, B′) and chick at E3 (Figure 3C), consistent with Pax3 transcription at these developmental stages. Deletion assays revealed that Motif1 (Figure 3D) was specifically required for CNE1 mediated transcription in the spinal cord of both zebrafish (Figure 3E, E′) and chick embryos (Figure 3F). By contrast, deletion of Motif3 (Figure 3J) reduced transgene expression throughout the AP axis of the zebrafish CNS (Figure 3K, K′) and extinguished enhancer activity in chick spinal cord (Figure 3L). Motif2 (Figure 3G) and Motif4 (Figure 3M) appeared to be dispensable in the context of these experiments (Figs. 3H–I and N–O).

Amongst the TFs that potentially interact with Motif3 (Table S2), the SoxB family represented the best candidates to promote the activity of CNE1 across the AP axis of the CNS. In agreement with several recent studies demonstrating the essential role of this family of TFs to promote enhancer activity in neural lineages [9], [10], point mutations within the putative HMG binding site of Motif3 reduced CNE1 activity in zebrafish and chick embryos (Figure S7). Moreover, examination of ChIP-Seq datasets produced in stem cell derived neuronal progenitors demonstrated that Sox3 and Sox11 directly bind CNE1, supporting a general positive input of SoxB proteins upon Pax3 expression in the CNS (data not shown) [41]. However, the input of this family of transcription factors is unlikely to explain the spatial restriction of CNE1 activity to the dorsal neural tube.

**Figure 4:**
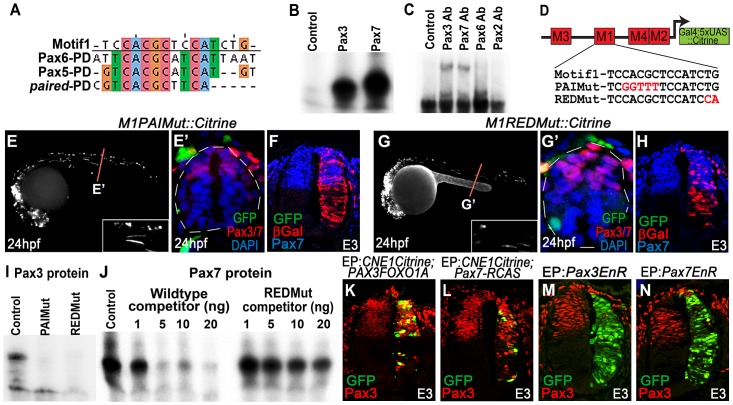
CNE1 mediates direct autoregulation and positive feedback via a paired domain binding site. (A) CNE1-Motif1 is homologous to the 5′ region of defined PD binding sites, however the alignment diverges in the 3′ region. (B) EMSA performed using Motif1 DNA and in-vitro synthesised Pax3 and Pax7 proteins, both of which can bind the sequence. (C) EMSA using Motif1 DNA and chick spinal cord nuclear extract, addition of antibodies against Pax3 or Pax7 to the reaction decreases the mobility of the DNA/protein complex. Addition of Pax6 or Pax2 antibodies does not alter the distribution of complexes within the EMSA. (D) Schematic illustrating the mutations targeted within CNE1-Motif1. (E, E′) *M1PAIMut* transgenic zebrafish exhibit a marked reduction in CNE1 activity in spinal cord progenitors, compared to the wildtype enhancer (n = 2/38, p<0.001). (F) Mutation of the PAI domain binding site precludes CNE1 activity in the chick neural tube (n = 0/8, p = 0.0007). (G, G′) Mutation of the RED domain binding site phenocopies Motif1 deletion in zebrafish embryos (n = 0/19, p<0.001) and chick embryos (H) (n = 1/5, p = 0.0128). (I) EMSAs performed using in vitro synthesised Pax3 protein and Motif1 DNA harboring either PAI or RED mutations. Mutation of either PAI or RED region precludes complex formation. (J) Competition EMSA performed using in vitro synthesised Pax7 protein, radiolabelled wildtype Motif1 DNA and non-labeled competitor probes. Non-labeled wildtype DNA effectively competes with radiolabelled probe for Pax7 binding, whereas DNA harboring the RED mutation cannot. (K) Electroporation of PAX3FOXO1A-RCAS induces ectopic CNE1 activity and Pax3 protein expression in the ventral neural tube (n = 9). (L) Pax7-RCAS electroporation also induces CNE1 activity and Pax3 expression (n = 7). Electroporation of dominant negative forms of Pax3 (M) (n = 7) or Pax7 (N) (n = 6) represses Pax3 protein expression within its endogenous domain.

Examination of the matrix represented by Motif1 revealed a 14 bp sequence similar to a Pax6 paired domain (PD) binding site (Table S2). We were particularly intrigued by this, as members of the Pax gene family have been shown to participate in selective auto- and inter-regulatory interactions [42]. Comparison of Motif1 consensus sequence with the defined Pax6 [43], Pax5 [44] and *paired* [45] PD binding sites revealed a high degree of homology towards the 5′ of the alignment, but poor consensus in the 3′ region (Figure 4A). Given these data and the described requirement for Motif1 in the dorsal spinal cord, we hypothesized this region could represent a PD binding site that exhibited specificity for Pax3 and its paralog Pax7.

We assessed the ability of both Pax3 and Pax7 to bind Motif1 by EMSA, using a 33 bp DNA probe and in vitro synthesized proteins. These assays revealed that both TFs interacted with this sequence, verifying it as a functional PD binding site (Figure 4B) Furthermore, supershift EMSAs using nuclear extracts from chick spinal cord and antibodies raised against selected Pax family members demonstrated the preferential occupation of Motif1 by the PD coded by Pax3/7 class genes (Figure 4C).

Previous binding and structural studies have shown that the PD is comprised of two helix-turn-helix subdomains, commonly termed PAI and RED, that interact with the 5′ and 3′ region of the binding site, respectively 46–48. The sequence of the PAI domain is largely conserved across the Pax gene family, whereas the variant RED domains have been proposed to underlie target site specificity [48]–[50]. We assessed the requirement of PAI and RED domain mediated Pax3/7 binding to Motif1 for CNE1 activity by targeting mutations within either half site, guided by the degree of conservation across this matrix (Figure 4D). A 5 bp substitution within the putative PAI half site resulted in a loss of CNE1 activity in the spinal cord progenitors of both zebrafish (Figure 4E, E′) and chick embryos (Figure 4F). Similarly, a 2 bp substitution within the putative RED domain half site extinguished the activity of this CRM in the dorsal neural tube of both model organisms (Figure 4G–H). In agreement with these in vivo observations, recombinant Pax3 protein was unable to bind Motif1 DNA probes carrying either the PAI or RED mutations in vitro (Figure 4I). Furthermore, competition EMSAs revealed the reduced ability of the RED mutant sequence to compete for Pax7 binding versus a wildtype probe (Figure 4J).

We next sought to assess the ability of Pax3 and Pax7 to induce CNE1 activity in the neural tube. Electroporation of a dominant active protein consisting of the DNA binding domain of human PAX3 fused to the transactivation domain of FOXO1A (PAX3FOXO1A) was sufficient to activate CNE1 mediated transcription in the ventral neural tube of chick embryos at E3 (Figure 4K). Moreover, PAX3FOXO1A was able to induce ectopic Pax3 protein expression (Figure 4K). Similarly, Pax7 electroporation was sufficient to induce ectopic Pax3 expression and CNE1 mediated transcription (Figure 4L). By contrast, electroporation of dominant-negative isoforms, constructed by fusion of the *engrailed* repressor domain to either Pax3 or Pax7 [51], reduced Pax3 expression within its endogenous domain at E3 (Figure 4M, N). Taken together, these findings support a model in which CNE1 functions to maintain Pax3 expression in the spinal cord by facilitating PD mediated autoregulation and positive feedback via Motif1.

## Discussion

In this study we provide evidence that the dynamic expression profile of Pax3 within the developing neural tube is achieved by the coordinated action of two distinct regulatory mechanisms, acting through separate CRMs. The combined activity of these enhancers converts transient inductive cues into a sustained domain of gene expression. CNE3 integrates inductive Wnt signaling and the repressive activity of HD transcription factors to initiate expression of Pax3 within the prospective dorsal neural tube. Subsequently, an autoregulatory loop acting via CNE1 is established that maintains Pax3 expression in the absence of activated Wnt signaling and HD mediated repression.

Previously, the zebrafish Pax3-GFP^I150^ BAC stable line has been shown to recapitulate the expression profile of *pax3a* in the spinal cord [52]. CNE1 and CNE3 are the only conserved enhancers contained within this genomic interval that exhibit specificity for the developing neural tube, suggesting that they are sufficient to induce and maintain Pax3 expression in this tissue. It is notable that the Pax3 locus contains an additional CRM located upstream of the promoter in higher vertebrate genomes that directs activity in the neural tube. However, this element is not phylogenetically conserved and is not required for gene expression in mice [22]–[25]. Thus, these data suggest that CNE1 and CNE3 represent the core regulatory circuit governing Pax3 expression in the CNS that has subsequently been further elaborated during vertebrate evolution.

The initiation of Pax3 transcription in response to Wnt signaling appears to be mediated through CNE3, a small highly conserved region of the genomic interval that has previously been identified as ECR2 and IR1 [22], [26]. This conclusion is supported by the observations that Tcf3 is bound to CNE3 in mouse embryonic stem cells and the requirement for Tcf/Lef sites for enhancer activity in zebrafish embryos [22], [26], [34]. However, the wide distribution of activated Wnt signaling within the posterior neural plate is inconsistent with the spatial restriction of Pax3 induction (Figure 2A′) [36]–[39]. Our analysis of CNE3 provides evidence that repression by HD proteins is essential to restrict the induction of Pax3 to the prospective dorsal neural tube. Nkx6.1 is able to directly bind CNE3 and mutation of a conserved HD binding motif results in ectopic enhancer activity. Moreover, the dorsal limit of Nkx6.1 expression coincides with the ventral limit of cells derived from the Pax3 lineage and gain-of-function experiments indicate that Nkx6.1 is sufficient to repress endogenous Pax3 protein expression. It should be noted that Nkx6.2 exhibits similar binding specificity to Nkx6.1 [53], [54] and is also expressed in the intermediate region of the neural plate and latterly the neural tube [14], [55]. Thus, a combination of these Nkx class repressors is likely to contribute to the establishment of the Pax3 expression domain during early CNS patterning.

The transcriptional activity of the Wnt pathway decreases in the neural tube as development progresses, as indicated by a downregulation of Wnt reporter transgene expression prior to the peak of Pax3 transcription in progenitors [36], [38], [39], [56]. Consistent with this, the activity of CNE3 diminished within the spinal cord over time. These data suggested that a separate enhancer is responsible for maintaining Pax3 expression in the neural tube and our analysis indicates that CNE1 is likely to fulfill this role. In support of this, CNE1 is the only Pax3 enhancer associated with p300 binding in CNS derived tissues at E11.5 (Figure S5) [33] and functional assays indicated that CNE1 remains active in the zebrafish and chick spinal cord after CNE3 activity has decreased.

Motif3 within CNE1, comprising Fox and Sox TFBS, is required for the activity of this enhancer across the AP axis of the CNS. The reduction of CNE1 activity in constructs carrying mutations in the HMG box binding site of Motif3, together with the identification of Sox11 and Sox3 binding at CNE1 in neural progenitors, supports a positive role for SoxB proteins on Pax3 expression and might account for the neural specificity of this enhancer [9], [10], [41]. More importantly, we provide evidence that direct autoregulation and positive feedback via a PD binding site is necessary for CNE1 activity in the spinal cord and that Pax3/7 bind this site in vitro and in neural cells. Furthermore, the activity of CNE1 and endogenous Pax3 expression is altered by misexpression or blockade of Pax3/7. Thus PD mediated autoregulation and positive feedback via CNE1 is likely to explain the maintenance of Pax3 expression in the neural tube.

**Figure 5:**
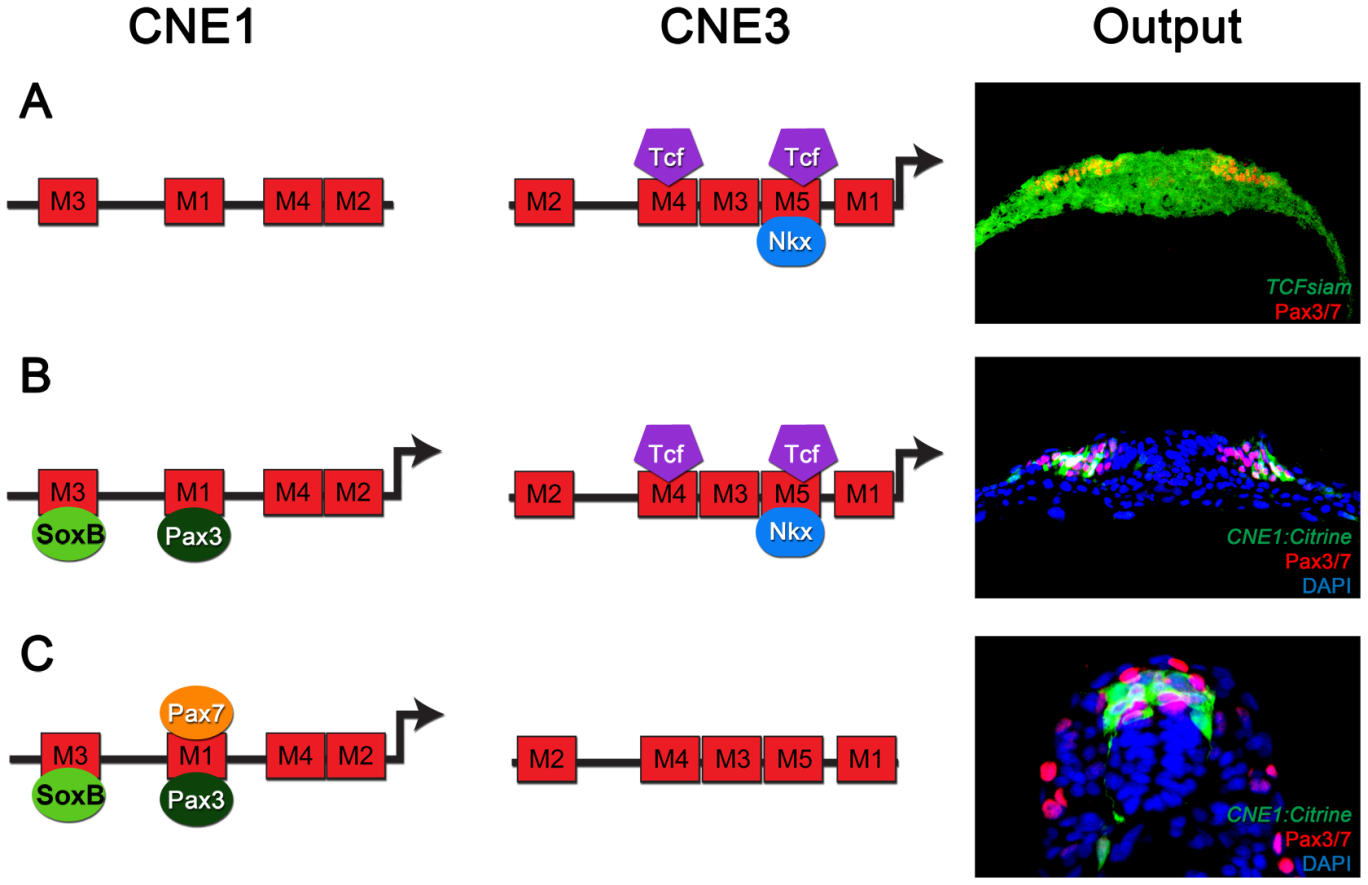
The regulatory logic of Pax3 expression in the neural tube. (A) Pax3 transcription in the developing CNS is induced by the binding of Wnt pathway effectors, such as Tcf3, to CNE3. Motif4 and Motif5 of CNE3 are likely to mediate this interaction as they contain phylogenetically conserved Tcf/Lef binding sites. The HD binding site within Motif5 is required to repress the activity of this enhancer in vivo. Consistent with this, Nkx6.1 binds to CNE3 in EMSAs and represses endogenous Pax3 expression in vivo. This combination of general activation and medial repression establishes the Pax3 expression domain in the lateral region of the neural plate. (B) Once induced, Pax3 protein binds Motif1 of CNE1 to mediate autoregulation. In addition to autoregulation, CNE1 activity might be restricted to neural tissue by SoxB transcription factors. At early stages of development, both CNE1 and CNE3 are transcriptionally active and may act synergistically to establish the Pax3 expression domain. (C) At later stages, when Pax3 expression reaches its maximum in the neural tube, the majority of dorsal progenitors do not experience active Wnt signaling. Furthermore, the Pax3 expression domain does not share a boundary with ventrally expressed Nkx family members at later stages of development. Our data suggests that a combination of autoregulation and Pax7 mediated positive feedback act to maintain Pax3 expression, seen in vivo as robust CNE1 activity in the absence of CNE3 mediated transcription.

Together, these data provide a molecular framework that describes the regulatory logic of Pax3 expression in the developing spinal cord. Expression is initiated by Wnt induction acting through CNE3, however this induction is limited to prospective dorsal regions of neural tissue by the activity of ventrally induced Nkx class proteins (Figure 5A). Pax3 protein expression then triggers a neural specific autoregulatory loop acting through CNE1 that secures transcription and removes the requirement for continued Wnt signaling and Nkx mediated ventral repression (Figure 5B and C). In addition, the induction of Pax7 expression at later developmental stages provides a means to augment and reinforce this maintenance loop (Figure 5C). Moreover, SoxB family proteins via a HMG box binding site in Motif3 (Figure 5 B and C) may limit CNE1 activity to neural tissue.

It is notable that at early developmental time points, both CNE1 and CNE3 appear to be active in Pax3 expressing cells. We propose they act in a cooperative manner to establish the Pax3 expression domain, thereby offering increased robustness and precision. For example, CNE1 mediated autoregulation could function not only to increase output from the Pax3 promoter but may also buffer fluctuations in CNE3 mediated transcription. This mechanism is reminiscent of the increased robustness and precision of gene expression achieved by the synergistic activity of multiple CRMs during *Drosophila* development [57]–[60]. In agreement with this hypothesis, a construct harboring genomic intervals containing both CNE1 and CNE3 has been shown to be more resistant to signaling pathway manipulation that either enhancer alone [26].

The acquisition of unique molecular identities within defined progenitor populations requires the translation of transient inductive cues into discrete expression domains. In the ventral neural tube this process is achieved using a transcriptional circuit involving cross-repression between TFs downstream of the ventral morphogen, Shh. This mechanism functions both to establish and maintain expression domains, as well as confer robustness to fluctuating levels of intracellular signaling [5]. In the case of Pax3, repression is used in combination with the inductive cue to establish the expression domain, but in contrast to the ventral neural tube, a direct autoregulatory loop plays a major role in maintaining expression. These two distinct functions are segregated between separate genomic elements. Thus, our findings expand the motifs employed within the neural tube GRN and highlight how differing regulatory mechanisms are manifest in the genome.

## Materials and Methods

### Comparative genomics and reporter plasmid construction

The genomic interval representing the Pax3 locus in each species was defined in the UCSC genome browser (http://www.genome.ucsc.edu/) and uploaded to the Mulan alignment suite [61], with appropriate repeat masking. Regions conserved with at least 65% identity over at least 40 bases in each input genome were selected for further study. The oligonucleotides listed in Table S3 were used to amplify CNEs from freshly prepared DKEY-20F20 BAC DNA (Genbank accession BX085193). Amplicons were subsequently inserted between the HindIII and SbfI sites upstream of a minimal thymidine kinase promoter in the MiniTol2 TKProm Gal4-5×UAS Citrine expression vector (Genbank accession KF545600), which exhibits no specific activity in control experiments (data not shown).

### Defining and manipulating putative binding sites

The Human sequence representing each CNE was used as the query sequence for cross-species BLAST searches, performed within the Ensembl genome browser (http://www.ensembl.org/). The alignment of enhancer sequences across vertebrate genomes was produced and analysed using ClustalW2 [62] and phylogenetically conserved motifs were defined using MEME [63]. TFBS within each motif were annotated using the TomTom tool of the MEME suite [64]. Targeted mutation of motifs and putative TFBS was performed using the Quickchange II XL site-directed mutagenesis kit (Stratagene) and the oligonucleotides listed in Table S3.

### Transgenic analysis in zebrafish, chick and mouse embryos

Zebrafish embryos were collected within 15 minutes of laying according to established procedures and injected with injected with a mixture of plasmid DNA (20 ng/µl) and Tol2 transposase mRNA (14 ng/µl). Embryos were maintained at 28.5°C and sorted on the basis of Citrine expression before fixation with 4% paraformaldehyde (PFA) at the desired stage. Chick assays were performed in Hamburger and Hamilton stage 11–13 embryos [65] by electroporation of reporter plasmid (500 ng/µl), Tol2 mRNA (14 ng/µl) and pCAGGS LacZ (1.5 µg/µl), according to described protocols [6]. DNA constructs contained within either the pCAGGS or RCAS expression vectors were electroporated at a concentration of 1.5–4 µg/µl. The PAX3FOXO1A-RCAS plasmid was created by subcloning the insert from the pCAGGS vector [66] using the oligonucleotides listed in Table S3. Embryos were maintained at 37°C and fixed with 4% PFA at the appropriate stage. Lineage tracing studies were performed by crossing Pax3Cre transgenic mice [27] with reporter strains that expressed YFP or both Tomato and GFP from the Rosa26 locus [67], the resulting embryos were fixed at the desired stage in 4% PFA.

### *In situ* hybridization, immunohistochemistry and imaging

Wholemount in situ hybridizations for *pax3a* (gift from Simon Hughes) were performed as described [68] before 14 µm transverse sections were prepared, when required. Analysis was carried out using a Zeiss Axiophot2 and Adobe Photoshop CS3. Antibody stainings of transverse sections of chick and mouse embryos were performed as previously described [6], [11]. The antibodies used for immunohistochemistry were rabbit anti-β-Galactosidase (ABD Serotec), mouse anti-Evx1 (DSHB), rabbit anti-GFP (Molecular Probes), sheep anti-GFP (Biogenesis), mouse anti-Nkx6.1 (DSHB), mouse anti-Pax3 (DSHB), and mouse anti-Pax7 (DSHB). Zebrafish Pax3/7 protein was visualized with DP312 (Gift from Nipam Patel), which was raised against the conserved homeodomain of *paired* [69]. DP312 has previously been shown to recognize both Pax3 and Pax7 in zebrafish embryos [70], [71]. Wholemount images were acquired using a Leica M205FA stereo-microscope and transverse sections were imaged using a Leica TCS SP2 confocal microscope. All images were processed with Adobe Photoshop CS3.

### Embryo analysis

Zebrafish experiments were analysed by determining the number of embryos labeled by Citrine expression in spinal cord progenitors as a proportion of the total transgenic population. The activity of enhancer constructs in the chick neural tube was assayed against the presence of β-Galactosidase antibody staining, which was used as an internal control of transgenesis. The statistical significance of transgenic assays was determined using two-tailed Fisher's exact tests.

### EMSAs

Radiolabelled DNA probes were produced as described in [72] using the oligonucleotides listed in Table S3. In vitro synthesized proteins were produced using the TnT coupled rabbit reticulocyte lysate system (Promega). Chick spinal cord nuclear extracts were prepared by manual tissue dissection, cell lysis in a buffer containing 100 mM HEPES, 15 mM MgCl2, 100 mM KCl and 1 M DTT and protein extraction in a buffer of 20 mM HEPES, 1.5 mM MgCl2, 0.42 M NaCl, 0.2 mM EDTA and 25% glycerol. Binding reactions were performed in a buffer of 4% Ficoll, 20 mM HEPES, 30 mM KCl, 1 mM DTT and 0.1 mM EDTA. Supershift reactions were performed using antibodies validated for use in chick tissue as described in [72].

## Supporting Information

Figure S1 Several functional CRMs are located within the 4^th^ intron of the Pax3 locus. (A) The full Mulan alignment of the Pax3 locus, summarised in Figure 1E. (B) CNE1 transient transgenics exhibit reporter expression across the AP axis of the developing CNS at 24 hpf (n = 67). (C) By contrast, CNE2 activity weakly labels postmitotic neurons within the dorsal spinal cord (n = 11). (D) At 24 hpf, CNE3 is active within the midbrain (mb), hindbrain (mb) and progenitors within the dorsal spinal cord (dP) (n = 51). (E) CNE4 is sufficient to direct transcription within muscle fibres (mf) and cranial neural crest (nc) (n = 32). (F) Surprisingly, CNE5 robustly labels the notochord (nc) and floor plate (fp) of the neural tube, tissues that do not express Pax3 at any point of their development (n = 34).
(TIF)

Figure S2 CNE1 activity recapitulates Pax3 expression. (A) CNE1 stable transgenic embryos assessed at 10 hpf exhibit Citrine expression in the developing hindbrain (hb) and presumptive dorsal progenitors (dP) within the lateral regions of the posterior neural plate. (B, B′) At 24 hpf, CNE1 activity recapitulates *pax3a* expression across the AP axis of the CNS and is restricted to the Pax3/7 domain of the dorsal spinal cord.
(TIF)

Figure S3 CNE1 is the only p300 bound Pax3 enhancer at E11.5. UCSC genome browser view displaying the binding profile of the enhancer associated transcription co-factor p300 within mouse limb, forebrain and midbrain tissue, prepared at E11.5 [33]. These data suggest that CNE1 is the only active enhancer within the Pax3 locus at this development stage and furthermore, that it is specifically active within midbrain derived tissue. These data are consistent with the activity profile of CNE1 and CNE3 in zebrafish and the expression of Pax3 within the mouse CNS at E11.5.
(TIF)

Figure S4 The conservation of CNE3 across vertebrates. ClustalW2 alignment of CNE3 across 12 vertebrate genomes, revealing multiple clusters of nucleotides that are conserved across the phyla. The location of motifs within CNE3, discovered by MEME analysis, is marked within the alignment.
(TIF)

Figure S5 Wnt pathway effectors directly bind CNE3. UCSC genome browser view displaying the binding profile of the Wnt pathway effector, Tcf3, across the Pax3 locus in mouse embryonic stem cells (mESC) [34]. These data demonstrate that CNE3 is bound by Tcf3, supporting the described role of the Wnt pathway in the initiation of Pax3 transcription.
(TIF)

Figure S6 The conservation of CNE1 across vertebrate genomes. ClustalW2 alignment of detailing the conservation of CNE1 across 12 vertebrate genomes. The location of statistically overrepresented motifs within this sequence is marked within the alignment.
(TIF)

Figure S7 Mutation of the HMG box site within Motif3 reduces CNE1 activity. (A) Schematic outlining the mutations introduced into the HMG box site of Motif3. *M3Mut1* transgenic zebrafish exhibit a reduction in CNE1 activity compared to controls (compare B to D) (n = 25/41, p<0.001). A similar reduction in CNE1 activity is observed in M3Mut2 transgenics (compare B to G) (n = 6/32, p<0.001). (F) Graphical summary of the effect of HMG binding site mutations upon CNE1 activity in zebrafish embryos. Experiments performed in chick reveal a reduction in CNE1 activity in both M3Mut1 (n = 3/7) and M3Mut2 (n = 2/5) electroporations, however this result did not reach statistical significance (compare C to E and H, respectively).
(TIF)

Table S1 Annotation of conserved TFBS within CNE3.
(DOCX)

Table S2 Annotation of conserved TFBS within CNE1.
(DOCX)

Table S3 List of oligonucleotides used in the study.
(DOCX)
