# Supplementary figures and images for: Distinct Regulatory Mechanisms Act to Establish and Maintain Pax3 Expression in the Developing Neural Tube
Source: PLoS Genet. 2013 Oct 3;9(10):e1003811. doi: 10.1371/journal.pgen.1003811 (PMC3789833; doi:10.1371/journal.pgen.1003811)

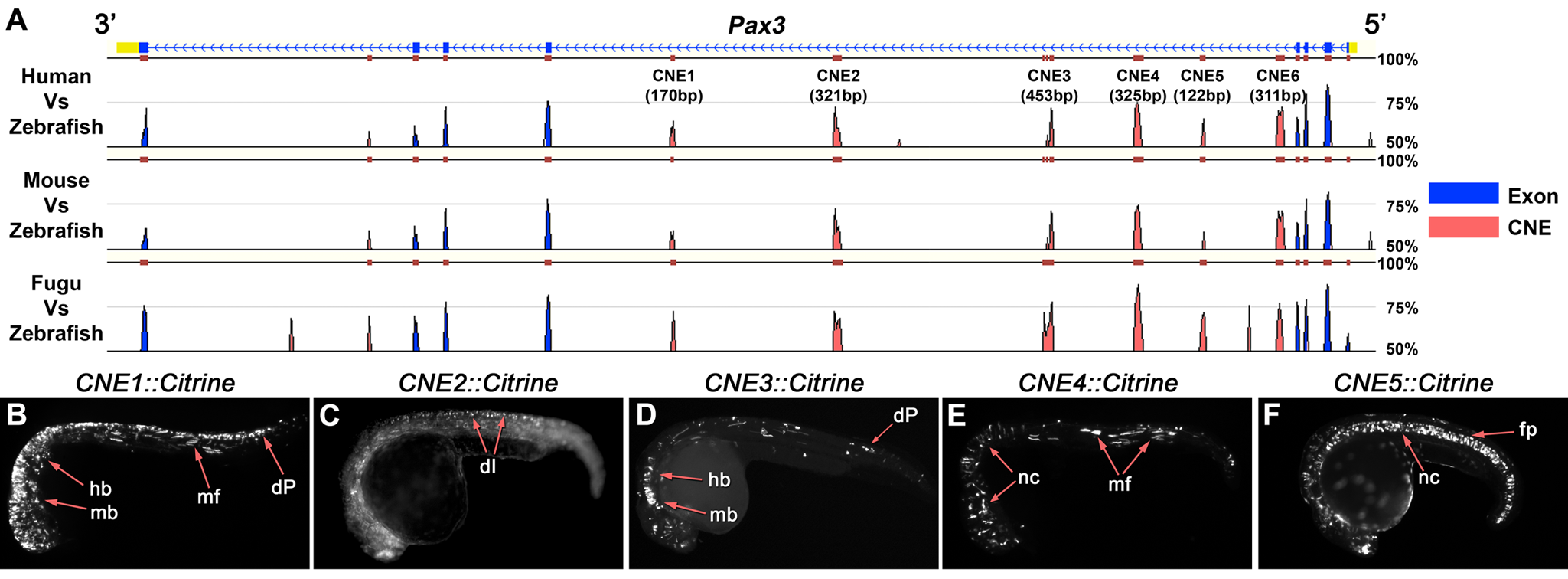

Supplement: Figure S1 — Several functional CRMs are located within the 4th intron of the Pax3 locus. (A) The full Mulan alignment of the Pax3 locus, summarised in Figure 1E. (B) CNE1 transient transgenics exhibit reporter expression across the AP axis of the developing CNS at 24 hpf (n = 67). (C) By contrast, CNE2 activity weakly labels postmitotic neurons within the dorsal spinal cord (n = 11). (D) At 24 hpf, CNE3 is active within the midbrain (mb), hindbrain (mb) and progenitors within the dorsal spinal cord (dP) (n = 51). (E) CNE4 is sufficient to direct transcription within muscle fibres (mf) and cranial neural crest (nc) (n = 32). (F) Surprisingly, CNE5 robustly labels the notochord (nc) and floor plate (fp) of the neural tube, tissues that do not express Pax3 at any point of their development (n = 34). (TIF) [file pgen.1003811.s001.tif]

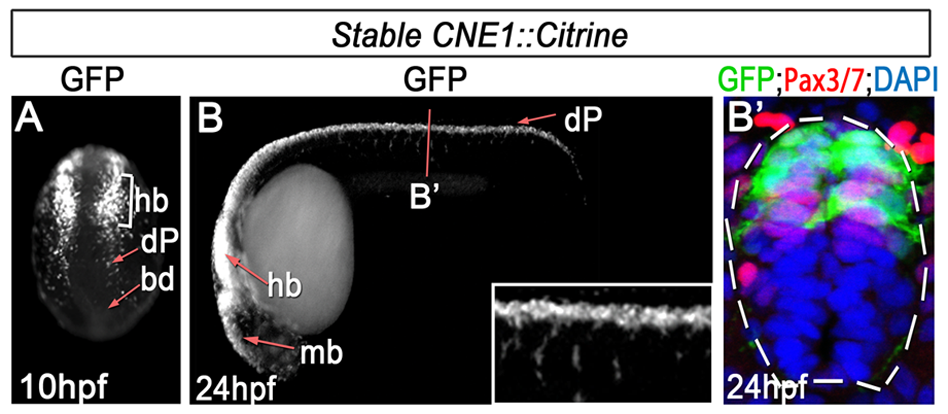

Supplement: Figure S2 — CNE1 activity recapitulates Pax3 expression. (A) CNE1 stable transgenic embryos assessed at 10 hpf exhibit Citrine expression in the developing hindbrain (hb) and presumptive dorsal progenitors (dP) within the lateral regions of the posterior neural plate. (B, B′) At 24 hpf, CNE1 activity recapitulates pax3a expression across the AP axis of the CNS and is restricted to the Pax3/7 domain of the dorsal spinal cord. (TIF) [file pgen.1003811.s002.tif]

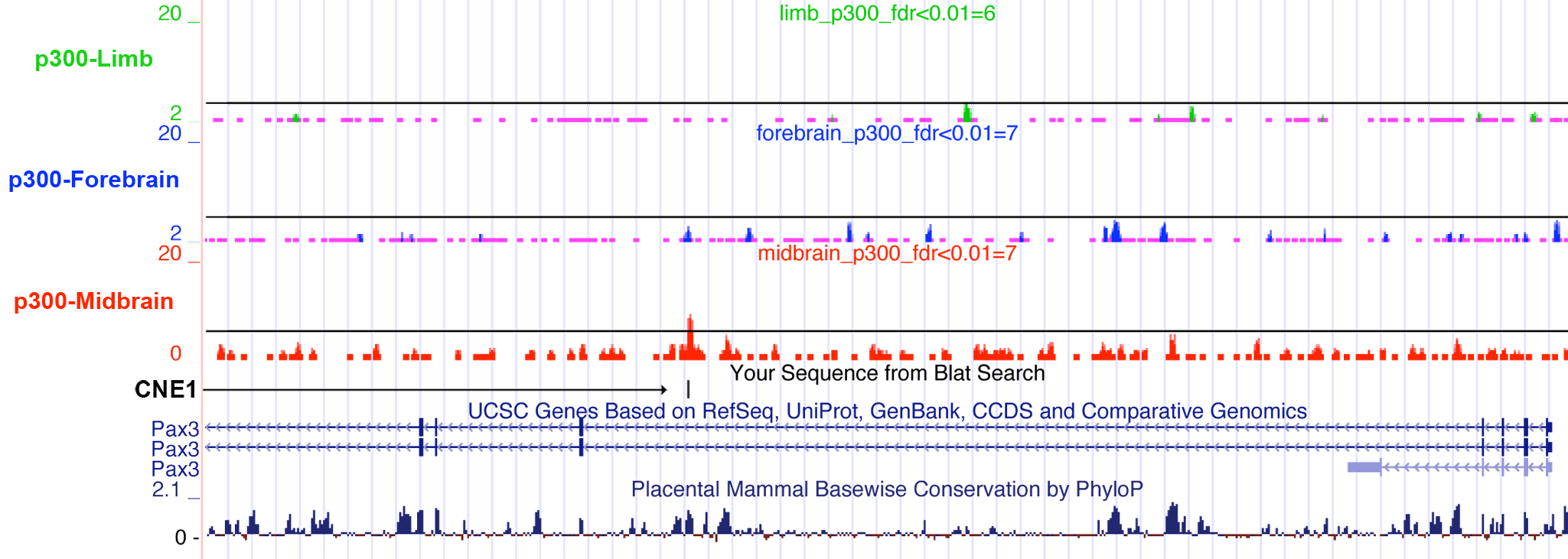

Supplement: Figure S3 — CNE1 is the only p300 bound Pax3 enhancer at E11.5. UCSC genome browser view displaying the binding profile of the enhancer associated transcription co-factor p300 within mouse limb, forebrain and midbrain tissue, prepared at E11.5 [33]. These data suggest that CNE1 is the only active enhancer within the Pax3 locus at this development stage and furthermore, that it is specifically active within midbrain derived tissue. These data are consistent with the activity profile of CNE1 and CNE3 in zebrafish and the expression of Pax3 within the mouse CNS at E11.5. (TIF) [file pgen.1003811.s003.tif]

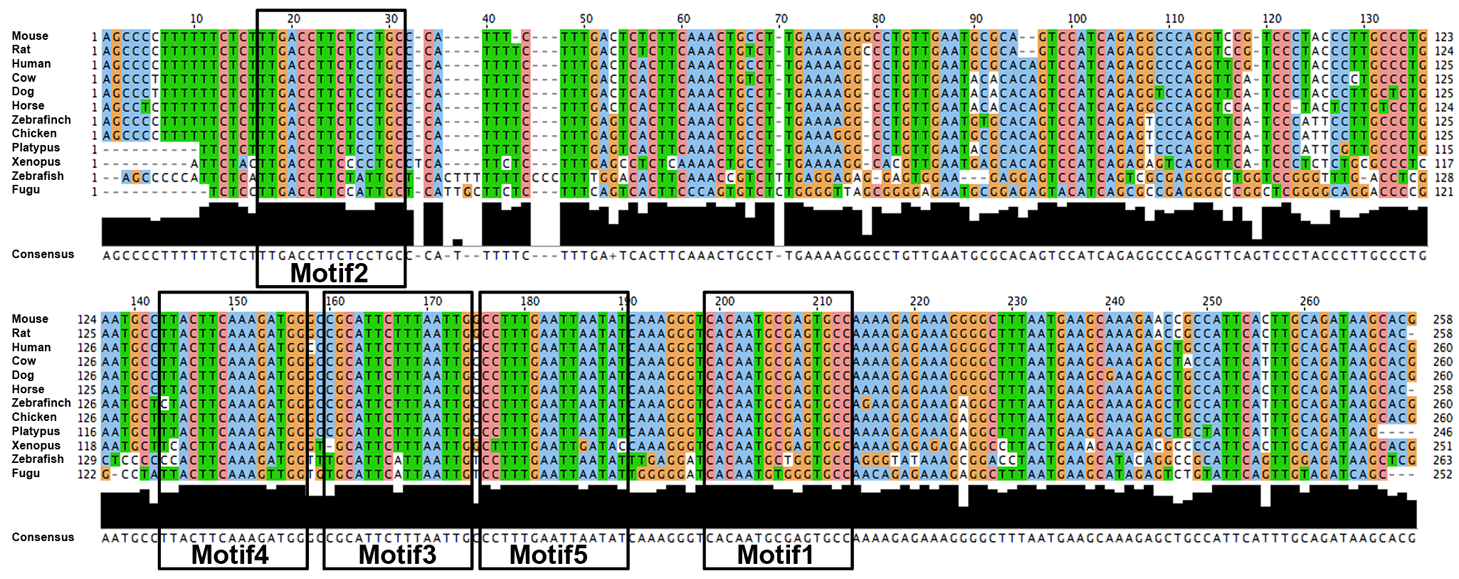

Supplement: Figure S4 — The conservation of CNE3 across vertebrates. ClustalW2 alignment of CNE3 across 12 vertebrate genomes, revealing multiple clusters of nucleotides that are conserved across the phyla. The location of motifs within CNE3, discovered by MEME analysis, is marked within the alignment. (TIF) [file pgen.1003811.s004.tif]

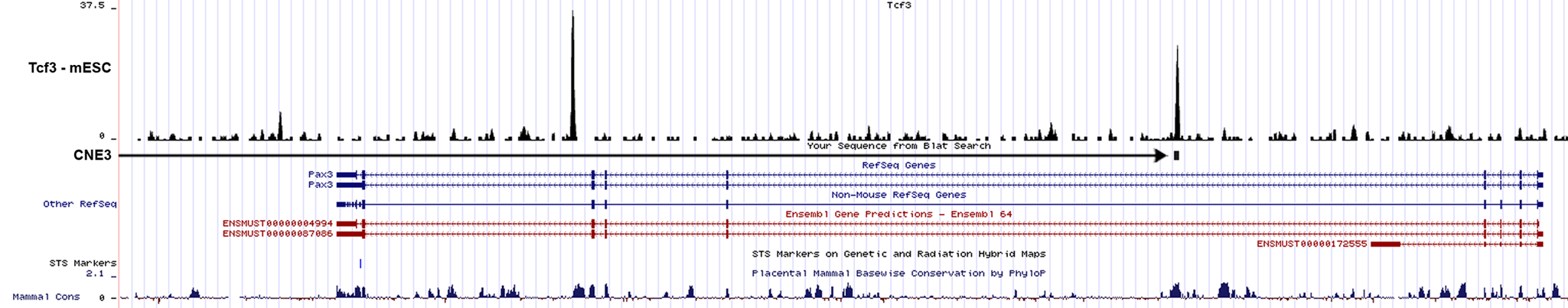

Supplement: Figure S5 — Wnt pathway effectors directly bind CNE3. UCSC genome browser view displaying the binding profile of the Wnt pathway effector, Tcf3, across the Pax3 locus in mouse embryonic stem cells (mESC) [34]. These data demonstrate that CNE3 is bound by Tcf3, supporting the described role of the Wnt pathway in the initiation of Pax3 transcription. (TIF) [file pgen.1003811.s005.tif]

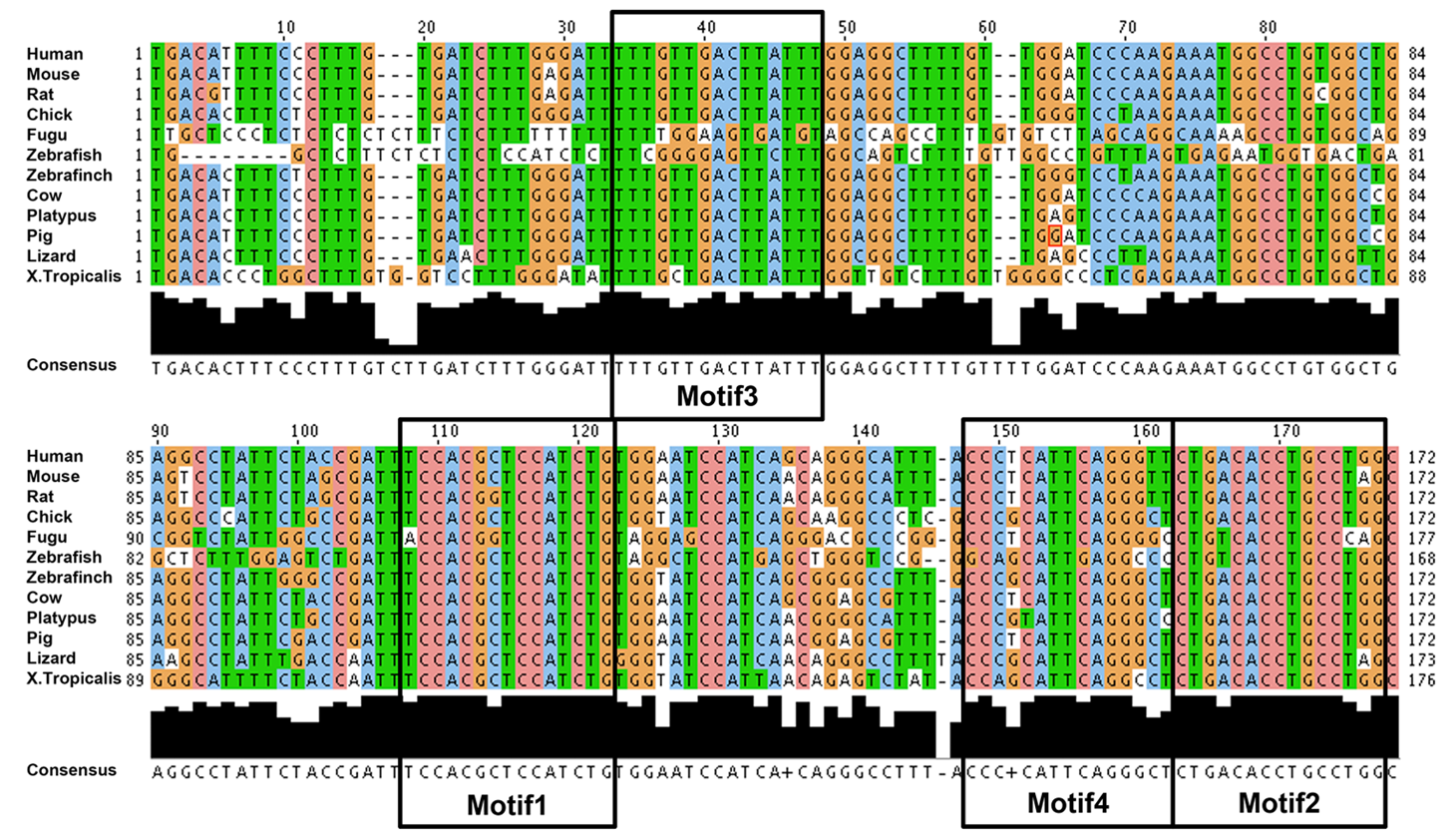

Supplement: Figure S6 — The conservation of CNE1 across vertebrate genomes. ClustalW2 alignment of detailing the conservation of CNE1 across 12 vertebrate genomes. The location of statistically overrepresented motifs within this sequence is marked within the alignment. (TIF) [file pgen.1003811.s006.tif]

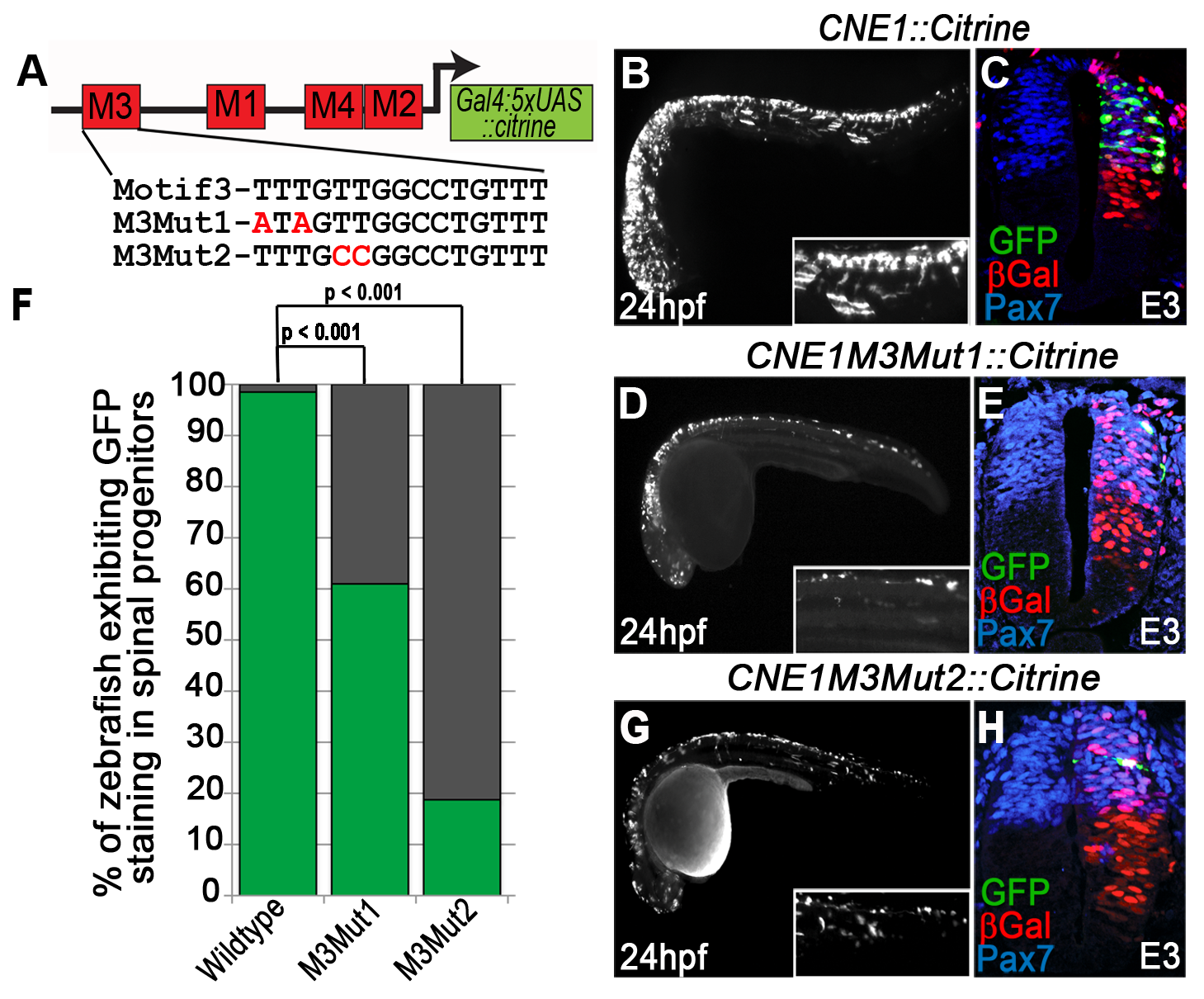

Supplement: Figure S7 — Mutation of the HMG box site within Motif3 reduces CNE1 activity. (A) Schematic outlining the mutations introduced into the HMG box site of Motif3. M3Mut1 transgenic zebrafish exhibit a reduction in CNE1 activity compared to controls (compare B to D) (n = 25/41, p<0.001). A similar reduction in CNE1 activity is observed in M3Mut2 transgenics (compare B to G) (n = 6/32, p<0.001). (F) Graphical summary of the effect of HMG binding site mutations upon CNE1 activity in zebrafish embryos. Experiments performed in chick reveal a reduction in CNE1 activity in both M3Mut1 (n = 3/7) and M3Mut2 (n = 2/5) electroporations, however this result did not reach statistical significance (compare C to E and H, respectively). (TIF) [file pgen.1003811.s007.tif]
