# Supplementary material for: Distinct Regulatory Mechanisms Act to Establish and Maintain Pax3 Expression in the Developing Neural Tube
Source: PLoS Genet. 2013 Oct 3;9(10):e1003811. doi: 10.1371/journal.pgen.1003811 (PMC3789833; doi:10.1371/journal.pgen.1003811)
Supplement: Table S1 — Annotation of conserved TFBS within CNE3. (DOCX) [file pgen.1003811.s008.docx]

**Table S1.** Annotation of conserved TFBS within CNE3.

| **Name** | **Logo (12 vertebrate genomes)** | **Location within CNE3 (zebrafish)** | **Matching Matrices identified by TomTom (JASPAR and UniPROBE)** |
| --- | --- | --- | --- |
| Motif1 | 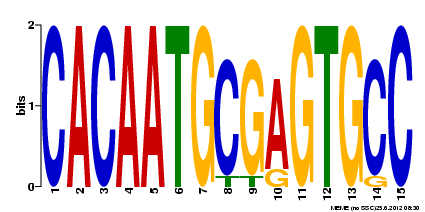 | Bases 382-396 | Gcm1, MATA1, Sox17, SOX10, Hbp1, Arid5a, h. |
| Motif2 | 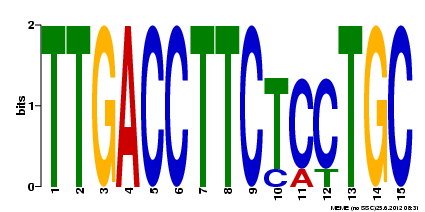 | Bases 206-220 | NR4A2, Esrrb, RXR::RAR, RORA, Klf4, tll, Nr2f2, INO2, Zfp187, INO4, opa, TEC1 |
| Motif3 | 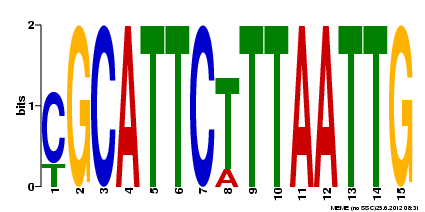 | Bases 343-357 | Hmx, CG34031, CG11085, Hmx3, unc-4, B-H2, YHP1, Hmx2, NK7.1, B-H1, CG13424, CG15696, tup, hbn, slou, Hmx1, SPT2, Cart1, Uncx4.1, CG32532, Phox2b, Nkx2-5, bsh, Alx4, Lhx9, Ubx, En1, Nkx6-3, C15, ARID3A, Hoxc5, Otp, Prrx2, Hbp1, Dbx2, Hoxb4, repo, Dr, unpg, Nkx6-1, Hoxc4, Arx, Prop1, CG32105, Vsx1, Hoxa7, inv, Shox2, Rx, HGTX, Pou3f2, PHDP, Lim1, CG7056, Lhx5, Lbx2, OdsH, Pou3f4, Dfd, abd-A, Vsx2, TEC1, Esx1, Rhox6, al, Msx1, ftz, CG11294., CG4328, Lhx1, Pou3f1, Pou1f1, Hoxb7, Dll, Phox2a, Sox1, CG9876, cad, Scr, bap, Lhx3, Lmx1a, Elf3, Antp, HOXA5, YOX1, Foxa2, Lim3, Hoxa4, Pou4f3, Awh, btn, Nobox, Hoxc6, lab. |
| Motif4 | 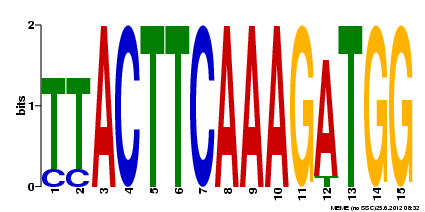 | Bases 326-340 | Vnd, Bbx, Tcf3, YY1, Tcf7, pan, Gata1, TGA1A, |
| Motif5 | 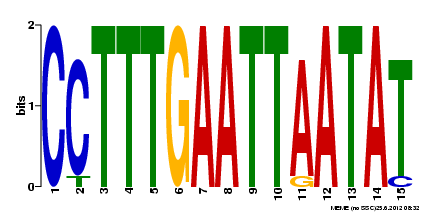 | Bases 359-373 | CG7056, Lmx1a, Lmx1b, Arid3a, Hbp1, Prrx2, Lhx5, RME1, YOX1, Phox2b, Lhx1, Uncx4.1, Sox1, Hoxa7, Prop1, Otp, Hoxb4, Alx4, Lhx9, Hoxa4, Tcf3, Tcf7, Lhx3, Ubx, Hoxb8, Lim1, al, Cart1, unc-4, Glis2, Hoxc4, Nkx6-1, CG11294, Nkx6-3, Vsx1, Hmx, hbn, repo, Isl2, Hoxc6, Ptx1, Lef1, Pax7, CG32105, abd-A, Msx1, CG15696, Zfp187, CG34031, pan, Tlx2. |
