# Supplementary material for: Distinct Regulatory Mechanisms Act to Establish and Maintain Pax3 Expression in the Developing Neural Tube
Source: PLoS Genet. 2013 Oct 3;9(10):e1003811. doi: 10.1371/journal.pgen.1003811 (PMC3789833; doi:10.1371/journal.pgen.1003811)
Supplement: Table S2 — Annotation of conserved TFBS within CNE1. (DOCX) [file pgen.1003811.s009.docx]

**Table S2.** Annotation of conserved TFBS within CNE1.

| **Name** | **Logo (12 vertebrate genomes)** | **Location within CNE1 (zebrafish)** | **Matching Matrices identified by TomTom (JASPAR and UniPROBE)** |
| --- | --- | --- | --- |
| Motif1 | 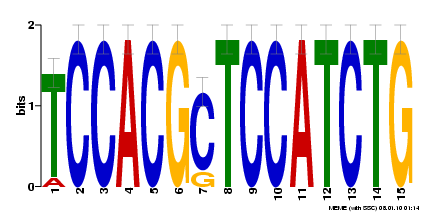 | Bases 99-114 | Hkb, Pax6, Arnt:Ahr, ZNF354C, Ascl2, HSF1, YY1, Irf6, SP1, Sp4, Gata1, Bcl6b, TAL1::TCF3, Tcfe2a, Zfp410, Snail, Bhlhb2 |
| Motif2 | 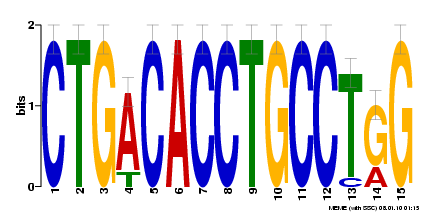 | Bases 152-167 | Six4, sna, ZEB1, ESR1, Esrra, CUP9, vis, PPARD, achi, ESR2 |
| Motif3 | 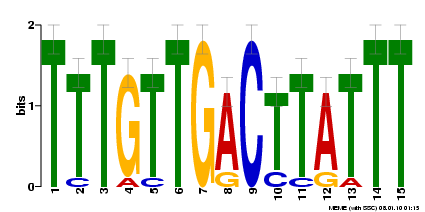 | Bases 48-63 | Foxa2, Foxj3, tll, Mafb, Foxk1, Foxl1, FOXD1, FoxA1, HCM1, fkh, FKH2, Foxj1, FKH1, br_Z4, Sox1, Mafk, Tcf1, RORA, Evi1, slp1, AP1, Yap7 |
| Motif4 | 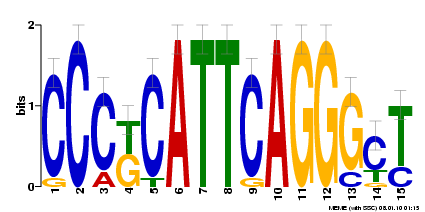 | Bases 139-154 | USV1, Sox17, REI1, TBF1, znf143, Hpb1, Sox8, Sox5, Rhox6, SOK2 |
