# Supplementary material for: Distinct Regulatory Mechanisms Act to Establish and Maintain Pax3 Expression in the Developing Neural Tube
Source: PLoS Genet. 2013 Oct 3;9(10):e1003811. doi: 10.1371/journal.pgen.1003811 (PMC3789833; doi:10.1371/journal.pgen.1003811)
Supplement: Table S3 — List of oligonucleotides used in the study. (DOCX) [file pgen.1003811.s010.docx]

**Table S3.** Oligonucleotides used in this study.

| **Oligo Name** | **Sequence (5’ to 3’)** |
| --- | --- |
| CNE1 FW | GGGCCCAAGCTTAGTGTTGCGTTTGGCTCTTT |
| CNE1 RE | CCAGGTGTCATTGAGCCTCTGGACGTCCGGGCCC |
| CNE2 FW | GGGCCCAAGCTTAAAGCCGCAACAACAAAGAG |
| CNE2 RE | TCCAGTCACGCTCTAGTCCAGGACGTCCGGGCCC |
| CNE3 FW | GGGCCCAAGCTTTTTCTCTCCCCTCCTTCACC |
| CNE3 RE | AAACCGTGCATCATTTCCACGGACGTCCGGGCCC |
| CNE4 FW | GGGCCCAAGCTTTCACCCCCAAAAATATCTGC |
| CNE4 RE | TGACAATCCTCGAGCCTCATGGACGTCCGGGCCC |
| CNE5 FW | GGGCCCAAGCTTACTGCCTTTTCCCAGACAAA |
| CNE5 RE | GTTTGCAGTGGGCTGATTCTGGACGTCCGGGCCC |
| CNE1M1deletion FW | GTGACTGAGCTCTTTGGAGTCTGATAGGCTCCATGAGCTGGGTCCGGG |
| CNE1M1deletion RE | CCCGGACCCAGCTCATGGAGCCTATCAGACTCCAAAGAGCTCAGTCAC |
| CNE1M2deletion FW | TGGGTCCGGGCAGCATTGAGGCCCACAATGAGGGCTGGACGCAGGGCA |
| CNE1M2deletion RE | TGCCCTGCGTCCAGCCCTCATTGTGGGCCTCAATGCTGCCCGGACCCA |
| CNE1M3deletion FW | TTTCGGGGAGTTCTTTGGCAGTCTTAGTGAGAATGGTGACTGAGCTCT |
| CNE1M3deletion RE | AGAGCTCAGTCACCATTCTCACTAAGACTGCCAAAGAACTCCCCGAAA |
| CNE1M4deletion FW | GTAGGCTCCATGAGCTGGGTCCGGCTGTCACCTGCCTGGCAGGGGGCG |
| CNE1M4deletion RE | CGCCCCCTGCCAGGCAGGTGACAGCCGGACCCAGCTCATGGAGCCTAC |
| CNE3M3deletion FW | GCTCCCCCCACTTCAAAGATGGTTTCCTTTGAATTAATATTTGAGGATCA |
| CNE3M3deletion RE | TGATCCTCAAATATTAATTCAAAGGAAACCATCTTTGAAGTGGGGGGAGC |
| CNE3M5deletion FW | AGATGGTTTGCATTCATTAATTGTTTGAGGATCACAATGCTGGTGCCAGG |
| CNE3M5deletion RE | CCTGGCACCAGCATTGTGATCCTCAAACAATTAATGAATGCAAACCATCT |
| CNE1Motif1PAIMut FW | TGGAGTCTGATTTCGGTTTTCCATCTGTAGGCTC |
| CNE1Motif1PAIMut RE | GAGCCTACAGATGGAAAACCGAAATCAGACTCCA |
| CNE1Motif1REDMut FW | GATTTCCACGCTCCATCCATAGGCTCCATGAG |
| CNE1Motif1REDMut RE | CTCATGGAGCCTATGGATGGAGCGTGGAAATC |
| CNE3Motif3HoxMut FW | AGATGGTTTGCATTCATTCCTTGTCCTTTGAATTAATA |
| CNE3Motif3HoxMut RE | TATTAATTCAAAGGACAAGGAATGAATGCAAACCATCT |
| CNE3Motif5HoxMut FW | CATTAATTGTCCTTTGAATTCCTATTTGAGGATCACAATGC |
| CNE3Motif5HoxMut FW | GCATTGTGATCCTCAAATAGGAATTCAAAGGACAATTAA |
| CNE1Motif1 EMSA FW | GAGTCTGATTTCCACGCTCCATCTGTAGGCTCC |
| CNE1Motif1 EMSA RE | GGAGCCTACAGATGGAGCGTGGAAATCAGACTC |
| CNE3Motif3-5 EMSA FW | CCACTTCAAAGATGGTTTGCATTCATTAATTGTCCTTTGAATTAATAT |
| CNE3Motif3-5 EMSA RE | ATATTAATTCAAAGGACAATTAATGAATGCAAACCATCTTTGAAGTGG |
| PAX3FOXOA1-RCAS FW | ACCACTGTGGCATCGATGTGCTGGTTGTTGTGCTGTC |
| PAX3FOXOA1-RCAS RE | CCGTACATCGCATCGATGGCTGCAGGAATTGGATATT |
